# Alteration of Introns in a Hyaluronan Synthase 1 (HAS1) Minigene Convert Pre-Mrna Splicing to the Aberrant Pattern in Multiple Myeloma (MM): MM Patients Harbor Similar Changes

**DOI:** 10.1371/journal.pone.0053469

**Published:** 2013-01-03

**Authors:** Jitra Kriangkum, Amanda Warkinton, Andrew R. Belch, Linda M. Pilarski

**Affiliations:** Department of Oncology, Cross Cancer Institute, University of Alberta, Edmonton, Alberta, Canada; Gentofte University Hospital, Denmark

## Abstract

Aberrant pre-mRNA splice variants of hyaluronan synthase 1 (HAS1) have been identified in malignant cells from cancer patients. Bioinformatic analysis suggests that intronic sequence changes can underlie aberrant splicing. Deletions and mutations were introduced into HAS1 minigene constructs to identify regions that can influence aberrant intronic splicing, comparing the splicing pattern in transfectants with that in multiple myeloma (MM) patients. Introduced genetic variations in introns 3 and 4 of HAS1 as shown here can promote aberrant splicing of the type detected in malignant cells from MM patients. HAS1Vd is a novel intronic splice variant first identified here. HAS1Vb, an intronic splice variant previously identified in patients, skips exon 4 and utilizes the same intron 4 alternative 3′splice site as HAS1Vd. For transfected constructs with unaltered introns 3 and 4, HAS1Vd transcripts are readily detectable, frequently to the exclusion of HAS1Vb. In contrast, in MM patients, HAS1Vb is more frequent than HAS1Vd. In the HAS1 minigene, combining deletion in intron 4 with mutations in intron 3 leads to a shift from HAS1Vd expression to HAS1Vb expression. The upregulation of aberrant splicing, exemplified here by the expression of HAS1Vb, is shown here to be influenced by multiple genetic changes in intronic sequences. For HAS1Vb, this includes enhanced exon 4 skipping and increased usage of alternative 3′ splice sites. Thus, the combination of introduced mutations in HAS1 intron3 with introduced deletions in HAS1 intron 4 promoted a shift to an aberrant splicing pattern previously shown to be clinically significant. Most MM patients harbor genetic variations in intron 4, and as shown here, nearly half harbor recurrent mutations in HAS1 intron 3. Our work suggests that aberrant intronic HAS1 splicing in MM patients may rely on intronic HAS1 deletions and mutations that are frequent in MM patients but absent from healthy donors.

## Introduction

It is becoming increasingly apparent that splicing defects play a key role in cancer, and that genomic changes in splicing elements [1]–[3], sometimes termed “splicing spoilers” [4], [5], can promote aberrant splicing. Because regulation of splicing is such a complex network [1], [4], all genetic variations in genomic DNA and pre-mRNA should be evaluated for their impact on splicing within any given genomic context. It has been estimated that ∼50% of mutations underlying genetic diseases cause aberrant splicing [6]. Alterations in a splicing site or splicing control region can have long range implications for splicing events, including altered 3-D architecture of pre-mRNA, activation of cryptic splice sites, exclusion of exons and/or inclusion of all or part of introns. Single mutations can strengthen otherwise weak splice sites and discriminate against otherwise strong splice sites [2]–[4]. Defective mRNA splicing caused by single nucleotide polymorphisms (SNPs) and/or splice site mutations often results in inactivation of tumor suppressor activity (e.g. HRPT2 [7], [8], PTEN [9], MLHI [10]–[12], ATR [13]) or generation of dominant negative inhibitors (e.g. CHEK2 [14], VWOX [15]). In breast cancer, aberrantly spliced forms of progesterone and estrogen receptors are found (reviewed in [2]). Intronic mutations inactivate p53 through aberrant splicing and intron retention, leading to the production of no or inactive p53 [16]. The large number of silent p53 genetic variations in cancer tend to be non-randomly located in exonic splicing enhancers, with a likely impact on p53 splicing [17], perhaps explaining their selection during oncogenesis and indicating that so-called “silent” mutations can have a profound impact on function.

In MM patients, we have identified a series of aberrant splice variants (Va, Vb and Vc) in the hyaluronan synthase 1 gene [18], [19]. These splice variants were found only in MM B cells, being undetectable in B cells from healthy donors. Alternative splicing of HAS1 involves exon 4 skipping (Va), partial intron 4 retention (Vc) or a combination of both (Vb). HAS1Vb expression correlates with significantly reduced survival in a cohort of MM patients [19]. Functional analysis of HAS1 minigene in transfectants shows that aberrant HAS1 splice variants gain anchorage independence and are transforming *in vivo* [20]. To determine the genetic basis for aberrant HAS1 splicing, the HAS1 gene from multiple cell types was sequenced for multiple patients, leading to identification of multiple exonic and intronic mutations, as well as SNPs, insertions/deletions and substitutions [21]. Although absent from healthy donors, a proportion of the newly identified HAS1 mutations were independently acquired in multiple unrelated patients, termed “recurrent”. Bioinformatic analysis predicts that a combination of novel mutations, SNPs and insertions/deletions in HAS1 direct the aberrant splicing that correlates with poor outcome [21], supporting the clinical relevance of genetic variations that lead to aberrant HAS1 splicing. However, splicing is a complex process and there are likely to be many combinations of genetic changes that can lead to aberrant splicing of HAS1.

In the present studies, we have introduced deletions and mutations into HAS1 constructs to identify some of the regions that influence aberrant intronic splicing, comparing the splicing patterns obtained in transfectants with those we detect in patients with MM. We find that introduced genetic variations in HAS1 constructs convert *in vitro* splicing patterns to the patterns seen *in vivo* in patients. We also find that genomic DNA from MM patients harbors novel recurrent mutations in HAS1 introns that appear to regulate aberrant splicing in transfectants. Our work suggests that aberrant intronic HAS1 splicing in MM patients relies on intronic HAS1 mutations that are frequent in MM patients but absent from healthy donors.

## Materials and Methods

### Ethics Statement

The study was approved by the Ethics Committee of the Cross Cancer Institute and University of Alberta. Written informed consent was provided in accordance with the Declaration of Helsinki.

### Plasmid Construction

HAS1FL (FLc) and HAS1g345 (G345) have been previously described [20], [21]. In brief, FLc is generated by cloning of HAS1 cDNA fragment into a mammalian expression vector pcDNA3 (Invitrogen). G345 is generated by replacing exons 3-4-5 cDNA sequence in FLc with the corresponding genomic DNA fragment.

Deletion constructs del5, del4, del3, del2 and del1 are derivatives of G345, being created by overlap extension PCR [22]. Two DNA subfragments were separately amplified: a) the 5′ piece extending from the beginning of the G345 construct to 680 bp downstream of exon 4, and b) the 3′ piece extending from the selected sequence in intron 4 to the end of the G345 construct. Overlapping ends were created by primer design. Joining of fragments was performed by mixing equimolar ratio of DNA fragments in standard polymerase chain reaction (PCR) using HiFi T*aq*DNA polymerase (Invitrogen) in the absence of primers and run for 7 cycles at 94°C for 30 s and 72°C for 4 min. The assembled fragment was further amplified in the presence of forward and reverse primers for 30 cycles, and then cloned into pcDNA3. The end products are constructs that have selective internal intron 4 deletion, each carried 680 bp of upstream intronic sequence joined to a specified downstream intronic sequence. These are 489 bp (del5), 361 (del4), 263 bp (del3), 198 bp (del2) and 84 bp (del1) sequences upstream of exon 5. The deleted protions are calculated to be 983 bp, 1111 bp, 1209 bp, 1274 bp and 1388 bp respectively.

Mutagenized HAS1 intron 3 (G1–28 m) was custom made by minigene synthesis (Mr.Gene). Constructs G345/G1–28 m and del1/G1–28 m were generated by overlap extension PCR of selected DNA fragments. For constructs carrying G1–18 m, G19–28 m, G19–24 m, G25–28 m or G27–28 m, DNA subfragments were amplified either from the parental construct or from the G1–28 m derivative, then joined together by overlap extension PCR. The accuracy of each construct was validated by DNA sequencing.

### Transient Expression and HAS1 Splicing Analysis

HeLa were originally obtained from the American Type Culture Collection (ATCC, Manassas, VA) and were grown at 37°C, 5% CO_2_ in DMEM (Invitrogen) supplemented with 10% fetal bovine serum (Invitrogen). Transfection was performed using Lipofectamine 2000 (Invitrogen) according to the manufacturer’s instructions. Twenty-four hours post-transfection, cells were washed twice with PBS and total RNA was prepared by Trizol reagent (Invitrogen) according to the manufacturer’s instructions. cDNA was reversed transcribed using dT15 and Superscript II (Invitrogen). PCR was run for 30 cycles at 94°C for 30 s, 60°C for 30 s, and 72°C for 30 s using Platinum T*aq* DNA polymerase (Invitrogen). PCR products were analyzed by agarose gel electrophoresis or DNA fragment analysis. For DNA fragment analysis, fluorescence primer was used and products were mixed with size standard in formamide and analyzed on an ABI Prism 3130*xl* Genetic Analyzer (Applied Biosystems). Data analysis was performed using GeneMapper software version 4.0. Primer sequences are summarized in Table 1.

**Table 1:** Summary of primer sequences.

Primer	Orientation	Sequence
E3	sense	5′GGGCTTGTCAGAGCTACT T3′
E5	antisense	5′AGGGCGTCTCTGAGTAGCAG 3′
E5I4	antisense	5′CTGGAGGTGTACCTGCACGGGGGC3′
5′Vb-specific	sense	5′GCGGTCCTCTAGAATCCTGCCCAG3′
5′outer SNPs	sense	5′TGTTCAGATCGGTTGCAGAGT3′
3′exon 4	antisense	5′CATGCACACACGCTAGGATA3′
HAS1seq5′	sense	5′GGGGTCTGTGCTGATCCTGG3′
HAS1seq3′	antisense	5′AACTGCTGCAAGAGGTTATTCC3′
β2m	sense	5′CCAGCAGAGAATGGAAAGTC3′
β2m	antisense	5′GATGCTGCTTACATGTCTCG3′

### Analysis of HAS1Vb/Vd Expression in Peripheral Blood Mononuclear Cells (PBMC)

Peripheral blood samples were collected from normal individuals and MM patients at diagnosis. MM was identified based on consensus criteria. Normal blood was obtained from University of Alberta Hospital emergency room as anonymous samples from 102 individuals selected as being over the age of 50 and without any obvious hematological issues.

PBMC were isolated by step gradient centrifugation (Ficoll-Paque Plus; GE Healthcare). RT-PCR followed *Transient expression and HAS1 splicing analysis* section, except that amplification was run for 35 cycles using E3/E5I4 primer set (Table 1) and PCR products were analyzed by DNA fragment analysis.

### Analysis of Recurrent Mutations in HAS1 Intron 3

Genomic DNA was prepared from PBMC using Trizol reagent (Invitrogen). HAS1 intron 3 region was amplified from 50 ng genomic DNA using 5′outer SNPs/3′exon4 primer set at 94°C for 30 s, 60°C for 30 s, and 72°C for 30 s for 35 cycles. The amplicon was treated with ExoSAP-IT reagent (USB) to remove excess primers and deoxyribonucleotides and subjected to direct sequencing using HAS1seq5′ or HAS1seq3′ primer and BigDye Terminator v3.1 (Applied Biosystems). Sequencing reaction was run on an ABI Prism 3130*xl* Genetic Analyzer (Applied Biosystems) and data were analyzed by DNA Sequencing Analysis Software v5.1 and SeqScape Software v2.5. Primer sequences are summarized in Table 1. Polymerase error rate in this study is shown to be less than 1 in 14,000 bp.

## Results

### 1. *In vitro* Analysis of Minigene Construct Characterizes Alternative Splicing of Human HAS1

**Figure 1:**
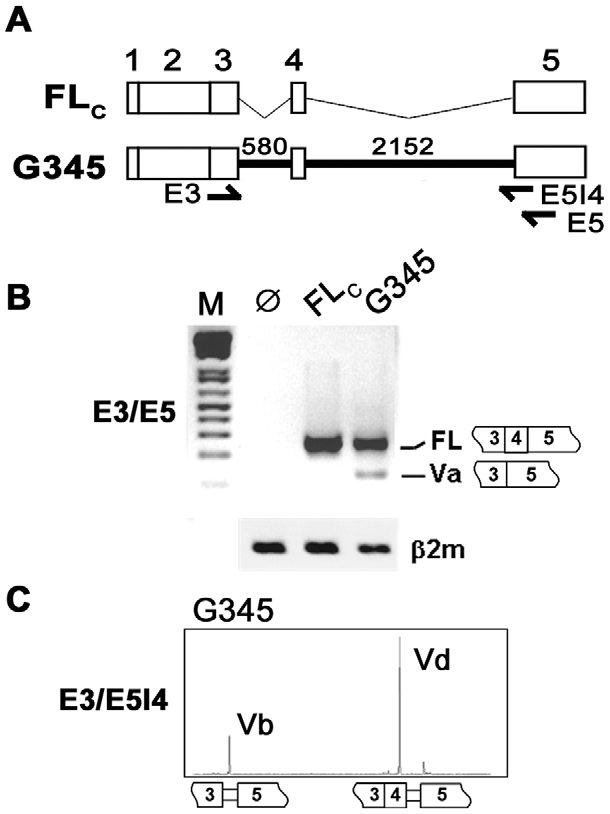
*In vitro* splicing analysis of human HAS1 minigene. Constructs FLc and G345 are shown in (A). Arrows show where PCR primers bind (E3, E5 and E5I4). The length of each intron in G345 is shown in bp. Each construct was transfected into HeLa cells and HAS1 splicing was studied by RT-PCR. Using E3/E5 primer set, products were analyzed by agarose gel electrophoresis (B). For E3/E5I4 primer set, amplicons were analyzed by DNA fragment analysis (C). Splice junctions for each product are also illustrated. Ø, mock transfection; β2m, control.

HAS1 splicing analysis has been established in a mammalian expression system where splicing products can be assessed by RT-PCR. Transient expression driven by the HAS1 minigene G345 construct (Figure 1A) yielded mainly full-length transcripts (FL) and varieties of alternatively spliced products similar to those found in *ex vivo* analysis [19] including a newly identified isoform termed HAS1Vd. Among HAS1 splice variants, Va (exon 4 skipped) is the most abundant, being detected along with FL on agarose gel when E3/E5 primer set was used (Figure 1B). Other variants are best determined by DNA fragment analysis using a selective primer set. HAS1Vb (exon 4 skipped and 59 bp downstream intron 4 retained) is of most interest due to its relevance in MM patients. Amplification by E3/E5I4 primer set predictably detected only HAS1Vb as E5I4 primer binds to exon 5/intron 4 junction. However, we always found another isoform, termed HAS1Vd, co-amplified with HAS1Vb, suggesting it is a common spliced product that has not been reported in the clinical studies (Figure 1C). Sequencing analysis showed that both Vb and Vd utilized the same alternative 3′SS that retained 59 bp of downstream intron 4 (−59): these two variants differed only in the inclusion (Vd) or exclusion (Vb) of exon 4 (133 bp). Overall, the splicing profile of G345 mimics normal HAS1 splicing and thus provides a model to study intronic sequence manipulation of the human HAS1 minigene.

### 2. Unlike HAS1Vb, the Expression of HAS1Vd is Comparable in HD or MM PBMC

Since HAS1Vd has not previously been reported, we evaluated its expression in PBMC of 102 healthy donors (HDs) and 93 MM patients. Using E3/E5I4 primer set in RT-PCR and DNA fragment analysis, we found that 9% of both populations expressed HAS1Vd, suggesting that HAS1Vd has little clinical relevance (Supplementary Tables S1 and S2). However HAS1Vb, documented previously as having clinical relevance, was found in 20% of unfractionated MM PBMC compared to 5% in HD PBMC, consistent with previous results [19]. Thus, MM PBMCs expressed HAS1Vb more frequent than Vd but HD PBMCs and transfectants expressed HAS1Vd more frequent than Vb, indicating that for the variants analyzed, splicing directed by the G345 construct is similar to that of HD and differs from that occurring in MM patients.

### 3. Partial Deletion of Intron 4 Increases Expression of HAS1Vd but not of HAS1Vb

**Figure 2:**
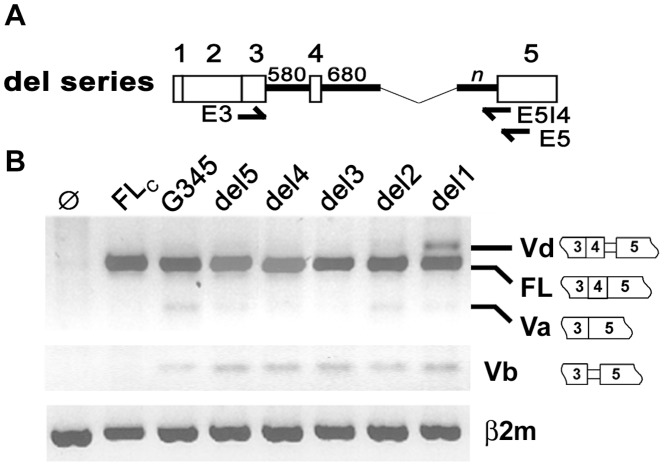
Partial deletion of intron 4 enhanced expression of HAS1Vd but not HAS1Vb. Selective portions of intron 4 were removed from G345 to create a series of del constructs as illustrated in (A). Each del construct carried 680 bp sequence of upstream intron 4 joined to the selective sequence in the downstream intron 4 (n). For del5, n = 489 bp; del4, n = 361 bp; del3, n = 263 bp; del2, n = 198 bp; del1, n = 84 bp. Arrows show where PCR primers bind (5′Vb primer is not shown in this diagram). Splicing of HAS1 in HeLa transfectants is shown in (B). RT-PCR was amplified by E3/E5 (top), 5′Vb/E5I4 (middle) or β2m primer set (bottom). Constructs FLc and G345 are illustrated in Figure 1. Ø, mock transfection.

Increased HAS1Vb was found to correlate with patient outcome in MM [19]. In MM and Waldenstrom’s macroglobulinemia (WM), we have identified recurrent mutations in HAS1 intron 4 [21], [23]. *In silico* analysis predicts that mutations and deletions in intron 4 can influence alternative splicing to use splice sites that generate HAS1Vb [21]. In this study, we determined if partial deletion of intron 4 is able to alter the splicing profile *in vitro*. A series of deletion constructs (del5-del1) was generated from G345, as mapped in Figure 2A. Deletion begins after 680 bp downstream of 5′SS and ends at variable distance upstream of 3′SS. Spliced isoforms produced by transfectants were characterized by RT-PCR on agarose gel electrophoresis and confirmed by DNA fragment analysis and sequencing of subclones. Figure 2B showed that expression driven by del5, del4, del3 and del2 were comparable to that of parental G345. Deletion beyond del 2 encouraged the use of alternative 3′SS (−59) since increased HAS1Vd was observed in del1. Thus, intronic sequence 198 bp upstream of exon 5 that is present in del2 is minimally required to mimic the splicing profile of the undeleted HAS1 minigene. This implies that the selected 1274 bp internal sequence could potentially be dispensable. In contrast, expression of HAS1Vb remains the same in all of the del1 transfectants (Figure 2B), suggesting that changes in intron 4 on its own, in the absence of enhanced exon 4 skipping, is not sufficient to promote aberrant HAS1Vb expression.

### 4. Mutagenesis of G-repeat Motifs in Intron 3 Enhances Exon 4 Skipping

**Figure 3:**
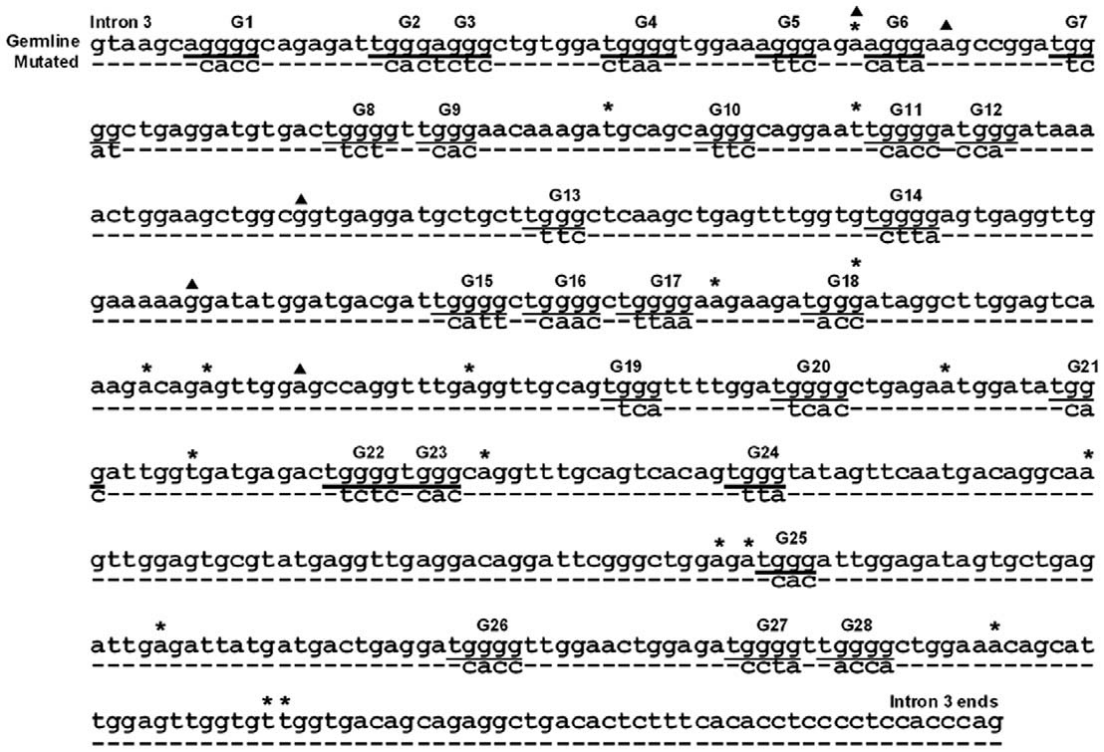
Site-directed mutagenesis of HAS1 intron 3 and recurrent mutations in MM. HAS1 intron 3 sequence is shown (A). (A/U)GGG repeats are underlined and numbered (G1, …, G28). The mutagenized sequence for each G motif is shown underneath. Asterisks (*) indicate the positions where recurrent mutations unique to MM PBMC were identified in the 50 MM patients reported here. Triangles (▴) represent recurrent mutations previously identified in 17 patients [21].

**Figure 4:**
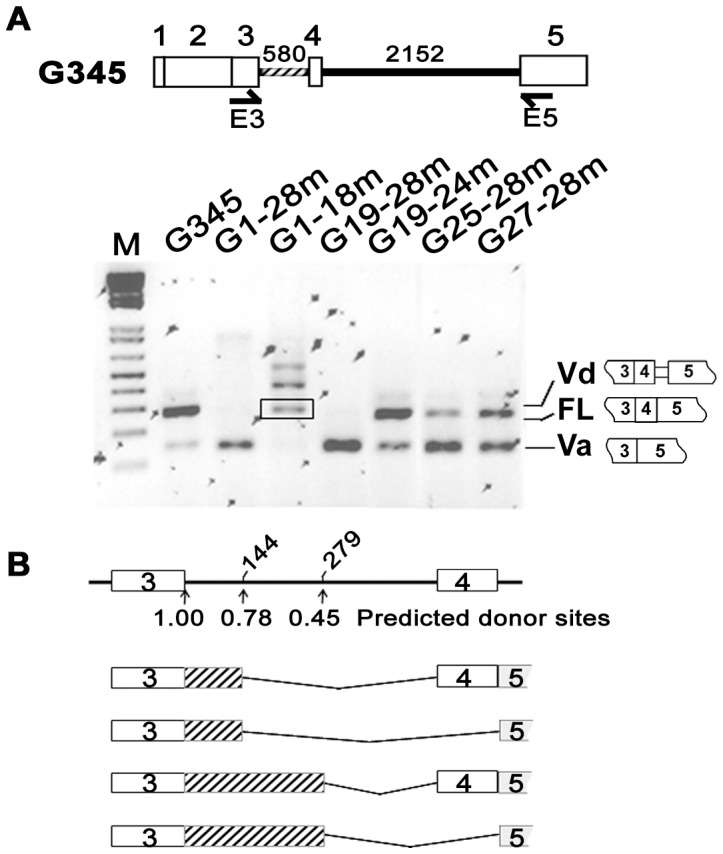
Mutagenesis of G-repeat motifs in HAS1 intron 3 enhances exon 4 skipping. Selected G-repeat motifs in G345 (striped line) were mutagenized according to sequences shown in Figure 3. Splicing profiles driven by various G345 derivatives were analyzed by RT-PCR using E3/E5 primer set and agarose gel electrophoresis (A). Product in box is not FL as determined by DNA fragment analysis (data not shown). Abnormal HAS1 transcripts driven by G345/G1–18 are summarized in (B). PCR products of G345/G1–18 m transfectants were cloned and spliced junctions were identified by sequencing of subclones. Arrows indicate authentic and cryptic donor sites which located 144 and 279 bp downstream of authentic donor site. The strength of each donor site is determined according to splice site prediction by a neural network (http://www.fruitfly.org/seq_tools/splice.html).

The sequence of HAS1 intron 3 (580 bp) is quite striking, comprising 28 repetitions of the motif (A/U)GGG (Figure 3). In addition, splicing enhancers and silencers are found within and around G-rich regions (Table 2). Site-directed mutagenesis of G-repeat motifs was studied to determine their roles in HAS1 splicing. Mutagenized sequences for each motif are shown in Figure 3. Splicing profiles driven by various mutagenized G345 constructs are summarized in Figure 4A. Here, we show that G-repeat motifs in HAS1 intron 3 play an important role in preventing exon 4 skipping. When all 28 G-repeat motifs were disrupted (G345/G1–28 m), the dominant splicing pattern (HAS1FL) was abolished, but splicing to generate HAS1Va was retained (Va>>FL). Less extensive mutagenesis, affecting only G1–18 (G345/G1–18 m) completely eliminated both FL and Va expression. This was replaced by multiple abnormal spliced products utilizing unconventional cryptic 5′ SS (Figure 4B). The complete loss of FL and Va in G345/G1–18 m could potentially be due to altered secondary structure since G345/G1–28 m (all motifs disrupted, including G1–18) still produced HAS1Va. Exon 4 skipping was most pronounced when only G19–28 repeats were mutagenized (G345/G19–28 m), in this case yielding only HAS1Va. More refined mutagenesis was studied to define the motifs within G19–28 that are most relevant to prevent exon 4 skipping. An elevated Va:FL ratio was observed among three constructs with mutagenized G19–24, G25–28 and G27–28. The highest Va:FL ratio was produced by G345/G25–28 m, followed by G345/G27–28 m and G345/G19–24 m and was consistent in replicate experiments (n = 5). This suggests that they all contribute to the inclusion of exon 4 but at variable degrees and are likely to work additively in this subregion.

**Table 2:** Splicing enhancers (ISE) and silencers (ESS) in the G-rich region of HAS1 intron 3.

Sequence	Type of element	References	Locations^1^
GGGGCTG	ISE (SF1)	[28], [34]	G7, G15, G16, G20, G28
GGGGTTGGGA	ESS	[35], [36]	G8–9, G11–12, G26,G27–28
GGGATGGGGT	ESS	[35]	G26, G27

1 Location of each G-motif is shown in Figure 3.

Altogether, this analysis showed that sequence modification of critical G- motifs appears to compromise the normal pattern of HAS1 expression by promoting increased exon 4 skipping.

### 5. Mutagenesis of G-repeat Motifs in Del1 Construct Promotes HAS1Vb Expression

Derivatives of del1 carrying mutagenized G-repeat motifs were studied in parallel to those of G345 carrying the same mutagenized motifs. Construct del1 serves as a model whereby both authentic and alternative 3′SS in intron 4 are frequently used to form HAS1FL or HAS1Vd (Figure 2B). Since HAS1Vb and HAS1Vd utilize the same alternative splice site, we asked if manipulation of G-repeat motifs in del1 would affect HAS1Vb expression.

**Figure 5:**
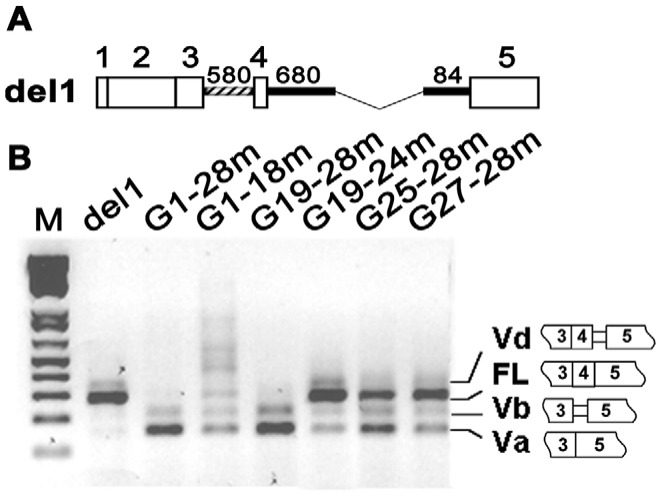
Mutagenesis of G-repeat motifs in del1 promotes HAS1Vb expression. Selected G-repeat motifs in del1 (striped line) were mutagenized according to sequences shown in Figure 3. Splicing profiles driven by various del1 derivatives were analyzed by RT-PCR using E3/E5 primer set and products were analyzed by agarose gel electrophoresis.

Splicing analysis of del1 derivatives is shown in Figure 5. Overall, this analysis showed that G-repeat motifs are important for the selection of splicing pathway, consistent with those found in G345 derivatives with the significant exception that all del1 derivatives gave rise to increased HAS1Vb expression; this did not occur for the G345 derivatives (Figure 4A). In del1/G1–28 m or del1/G19–28 m, HAS1FL expression was almost eliminated and splicing to form HAS1Va and HAS1Vb became dominant. In del1/G1–18 m, multiple aberrant splicing events predominated over the frequent variants usually detected, in line with those observed in G345/G1–18 m. Splicing was least disturbed in del1/G19–24 m. Exon 4 skipping driven by del1/G25–28 m was more pronounced than that by del1/G27–28 m, in both cases yielding increased expression of HAS1Va and HAS1Vb when compared to parental del1. Our study thus demonstrated that aberrant HAS1Vb splicing could be enhanced by combining genetic manipulation events that lead to increased exon 4 skipping with genetic manipulations that enable increased usage of alternative 3′SS (−59).

### 6. Recurrent Genetic Variations in HAS1 Intron 4 and Recurrent Mutations in HAS1 Intron 3 are Frequent in MM Patients

Since genetic changes in intron 3 and 4 promote changes in aberrant splicing that favor generation of HAS1Vb (Figure 5), we asked whether genetic variations similar to those created in transfectants are found in genomic HAS1 of MM patients. Although genetic changes throughout the genome potentially influence local splicing patterns, it was our working hypothesis that mutations distributed within intron 3 may play a significant role. Our initial studies identified 41 recurrent mutations (genetic variations that are shared by 2 or more unrelated patients) in MM patients but not in HD [21]. Of these, 24 recurrent mutations were found in intron 4 and five in intron 3 (marked by ▴ in Figure 3), recurrent in 2–14 of the 17 patients analyzed. In the present study, HAS1 intron 3 was sequenced from a second group of 50 MM patients. For 22/50 patients, 18 recurrent mutations unique to MM were identified (a>G = 12, t>C = 5, g>A = 1, marked by * in Figure 3); a significant proportion of these intron 3 mutations were also found in the earlier study but at that point were still presumed to be unique [21]. Individual mutations were recurrent in 2–7 patients. Among these, 17/18 recurrent mutations increased the G-C content of intron 3 and 6/18 either created or disrupted G runs in intron 3. This demonstrates that mutations frequently occurring in MM patients are located near those introduced to a construct by *in vitro* mutagenesis. By extrapolation, these intron 3 mutations in MM patients may contribute to aberrant splicing of HAS1 in malignant cells from patients, where HAS1Vb is more frequent than HAS1Vd.

## Discussion

In this work we show that mutations and deletions in introns 3 and 4 of HAS1 can alter pre-mRNA splicing events to promote aberrant splicing of the type detected in malignant cells from patients with MM. Among splice variants, although HAS1Va (exon 4 skipped) is common, HAS1Vb (exon 4 skipped and 59 bp downstream intron 4 retained) appears to be clinically more relevant because in MM, its overexpression correlates with the worst clinical outcome. Since aberrant splicing involves exons 3-4-5, it seems likely that frequent mutations in introns 3 and 4 may be involved in the selection of splice sites for pre-mRNA splicing. We utilized HAS1 minigene transfection to evaluate splicing profiles. A newly identified intronic splice variant, HAS1Vd, utilizes an otherwise cryptic splice site in intron 4 to generate a transcript including a segment of intron 4 and encoding a truncated protein. For constructs with unaltered introns 3 and 4, HAS1Vd transcripts are readily detectable, frequently to the exclusion of HAS1Vb which utilizes the same intron 4 splice site. In contrast, HAS1Vb is detected more frequently in patient cells where HAS1Vd is infrequent. For nearly half of MM patients, HAS1Vb is expressed in the MM clone at the time of diagnosis [19], [21]. For patients lacking HAS1 splice variants at diagnosis, these transcripts were often detected at later stages of disease [19]. Analysis of a series of directed deletions in HAS1 intron 4 showed that splicing of HAS1Vd could be elevated, but HAS1Vb remained unaffected, despite their use of the same 3′ splice site in intron 4. Thus, changes in intron 4 alone were insufficient to promote the splicing pattern observed in patients. Combining deletion in intron 4 with mutations in intron 3 however resulted in skipping of exon 4 and promotion of the splicing pattern that leads to a shift from HAS1Vd expression to HAS1Vb expression, the pattern observed in malignant cells from MM patients. To determine the relevance of these genetic changes *in vivo*, we sequenced intron 3 from genomic DNA of MM PBMC. Consistent with the influence on HAS1Vb of changes made by site directed mutagenesis, in almost half of MM patients analyzed, we found recurrent mutations in intron 3, some located proximate to G repeats as well as some that increased the GC content and increased or decreased the number of G repeats. Previous work has shown that essentially all MM patients analyzed harbored genetic variations in intron 3 and intron 4 [21]. These observations are consistent with the idea that in MM patients, genetic variations in introns 3 and 4 alter splice site selection resulting in intronic splice variants. Together, these promote use of alternative splice sites to generate intronic splice variants that skip exon 4, operationally resulting in loss of HAS1Vd splicing and enhanced expression of the clinically relevant HAS1Vb variant.

Deletion analysis of intron 4 was aimed at identifying an intronic region that is important for aberrant splicing of HAS1. Mutations previously identified in MM and WM are frequent in the two “T” stretches and TTTA repeats of intron 4 [21]. The first T stretch was removed from deletion construct del5 while both T stretches were deleted from del4. For del3 and other smaller del constructs, the two T stretches and TTTA repeats were altogether eliminated. Our splicing analysis showed that there was no remarkable change in the splicing profile whether these motifs are present or not, provided that minimum 198 bp sequence (del2) flanking the authentic 3′SS remains undisturbed (Figure 2). While *in silico* analysis showed that these mutations are important to the formation of HAS1Vb [21], *in vitro* splicing analysis did not detect increased expression of HAS1Vb even when the usage of relevant alternative 3′SS was increased. Thus, frequent mutations in the common motifs of HAS1 intron 4 may contribute to aberrant splicing in ways that are beyond the scope of this analysis. Recent epigenetics studies supported the idea that total intronic length could contribute to aberrant splicing via regulation of transcription rate, chromosomal structure and histone modification [24].

G-repeat motifs make up 75% of intron 3 sequences, thus prompting us to study their influence on HAS1 splicing. Intronic G repeats have been shown to modulate splicing in several genes for several species [25]–[27]. In α-globin intron 2, G triplets acted additively both to enhance splicing and to facilitate recognition of exon-intron borders [28]–[30]. Likewise, six (A/U)GGG motifs acted additively in IVSB7 of chicken β-tropomyosin and were essential to spliceosome formation [31]. In human thrombopoietin, intronic G repeats work in a combinatorial way to control the selection of the proper 3′SS; binding to hnRNP H1 is critical for the splicing process as removal of hnRNP H1 could promote the usage of the cryptic 3′ SS [32]. Our mutagenesis studies showed that modification of G-repeat motifs in HAS1 intron 3, especially the last 2–4 motifs of downstream sequence (G25–28 or G27–28), was sufficient to enhance exon 4 skipping (Figure 4). Mutagenesis of intron 3 G-repeat motifs, when combined with an increased usage of alternative 3′SS (−59) caused by intron 4 deletions resulted in an increased HAS1Vb expression (Figure 5). This indicates that the upregulation of aberrant splicing, exemplified here by the expression of HAS1Vb, is influenced by multiple genetic changes in intronic sequences. For HAS1Vb, this includes enhanced exon 4 skipping and increased usage of alternative 3′SS.

Provocatively, we find that genomic DNA from MM patients harbors novel recurrent mutations in HAS1 intron 3 and/or intron 4 that are similar to those in the mutagenized HAS1 minigene constructs we introduced to transfectants. In transfectants, the introduction of altered constructs carrying introduced mutations in HAS1 intron 3 and introduced deletions in HAS1 intron 4 promoted a shift to an aberrant splicing pattern already identified as being clinically significant in patients with MM [21], [33]. Most MM patients harbor genetic variations in intron 4 [21]. Nearly half of MM patients express HAS1Vb at diagnosis [19] and as shown here, nearly half harbor recurrent mutations in HAS1 intron 3. Our work suggests that aberrant intronic HAS1 splicing in MM patients relies on intronic HAS1 mutations that are frequent in MM patients but absent from healthy donors. Our previous work, coupled with the molecular analysis reported here, suggests that the splicing regions in introns 3 and/or 4 might represent druggable targets to prevent aberrant HAS1 splicing.

## Supporting Information

Table S1 **Expression of HAS1Vb and Vd in MM PBMC.**
(DOC)

Table S2 **Expression of HAS1Vb and Vd in HD PBMC.**
(DOC)
