# Correction: Alteration of Introns in a Hyaluronan Synthase 1 (HAS1) Minigene Convert Pre-mRNA Splicing to the Aberrant Pattern in Multiple Myeloma (MM): MM Patients Harbor Similar Changes

**DOI:** 10.1371/annotation/9984da27-0e7f-4c7f-bcd3-bef1a4c0be87

**Published:** 2013-04-30

**Authors:** Jitra Kriangkum, Amanda Warkentin, Andrew R. Belch, Linda M. Pilarski

In the Title, "Pre-Mrna" should be "Pre-mRNA"

The second author's name was misspelled. The correct name is: Amanda Warkentin. 

The correct citation is:

Kriangkum J, Warkentin A, Belch AR, Pilarski LM (2013) Alteration of Introns in a Hyaluronan Synthase 1 (HAS1) Minigene Convert Pre-Mrna Splicing to the Aberrant Pattern in Multiple Myeloma (MM): MM Patients Harbor Similar Changes. PLoS ONE 8(1): e53469. doi:10.1371/journal.pone.0053469 

