# Supplementary material for: Alteration of Introns in a Hyaluronan Synthase 1 (HAS1) Minigene Convert Pre-Mrna Splicing to the Aberrant Pattern in Multiple Myeloma (MM): MM Patients Harbor Similar Changes
Source: PLoS One. 2013 Jan 3;8(1):e53469. doi: 10.1371/journal.pone.0053469 (PMC3536762; doi:10.1371/journal.pone.0053469)
Supplement: Table S1 — Expression of HAS1Vb and Vd in MM PBMC. (DOC) [file pone.0053469.s001.doc]

**Table S1** Expression of HAS1Vb and Vd in MM PBMC

| **ID** | **Vb** | **Vd** |
| --- | --- | --- |
| MM1 | - | - |
| MM2 | - | - |
| MM3 | - | - |
| MM4 | + | + |
| MM5 | - | - |
| MM6 | - | - |
| MM7 | - | + |
| MM8 | - | - |
| MM9 | - | - |
| MM10 | - | - |
| MM11 | + | - |
| MM12 | + | - |
| MM13 | + | + |
| MM14 | - | - |
| MM15 | - | - |
| MM16 | - | - |
| MM17 | - | - |
| MM18 | - | - |
| MM19 | - | - |
| MM20 | - | - |
| MM21 | - | + |
| MM22 | - | - |
| MM23 | - | - |
| MM24 | - | - |
| MM25 | - | - |
| MM26 | - | - |
| MM27 | - | - |
| MM28 | - | - |
| MM29 | - | - |
| MM30 | - | - |
| MM31 | - | - |
| MM32 | - | - |
| MM33 | + | - |
| MM34 | - | + |
| MM35 | - | - |
| MM36 | - | - |
| MM37 | - | - |
| MM38 | - | - |
| MM39 | - | - |
| MM40 | + | - |
| MM41 | - | - |
| MM42 | + | - |
| MM43 | - | - |
| MM44 | - | - |
| MM45 | - | - |
| MM46 | + | - |
| MM47 | - | - |
| MM48 | - | - |
| MM49 | + | - |
| MM50 | - | - |
| MM51 | - | - |
| MM52 | - | - |
| MM53 | - | - |
| MM54 | - | - |
| MM55 | - | + |
| MM56 | - | - |
| MM57 | + | - |
| MM58 | - | - |
| MM59 | - | - |
| MM60 | - | - |
| MM61 | - | - |
| MM62 | - | - |
| MM63 | - | - |
| MM64 | - | - |
| MM65 | - | - |
| MM66 | - | - |
| MM67 | - | - |
| MM68 | - | + |
| MM69 | - | - |
| MM70 | - | - |
| MM71 | - | - |
| MM72 | + | - |
| MM73 | - | - |
| MM74 | - | - |
| MM75 | - | - |
| MM76 | - | + |
| MM77 | - | + |
| MM78 | - | - |
| MM79 | - | - |
| MM80 | - | - |
| MM81 | - | - |
| MM82 | + | - |
| MM83 | + | - |
| MM84 | - | - |
| MM85 | + | - |
| MM86 | + | - |
| MM87 | - | - |
| MM88 | - | - |
| MM89 | + | - |
| MM90 | + | - |
| MM91 | + | - |
| MM92 | + | - |
| MM93 | - | - |
