# Supplementary material for: Alteration of Introns in a Hyaluronan Synthase 1 (HAS1) Minigene Convert Pre-Mrna Splicing to the Aberrant Pattern in Multiple Myeloma (MM): MM Patients Harbor Similar Changes
Source: PLoS One. 2013 Jan 3;8(1):e53469. doi: 10.1371/journal.pone.0053469 (PMC3536762; doi:10.1371/journal.pone.0053469)
Supplement: Table S2 — Expression of HAS1Vb and Vd in HD PBMC. (DOC) [file pone.0053469.s002.doc]

**Table S2** Expression of HAS1Vb and Vd in HD PBMC

| **ID** | **Vb** | **Vd** |
| --- | --- | --- |
| N1 | - | + |
| N2 | - | - |
| N3 | - | - |
| N4 | - | + |
| N5 | - | - |
| N6 | - | - |
| N7 | - | - |
| N8 | - | - |
| N9 | - | - |
| N10 | - | - |
| N11 | - | - |
| N12 | - | - |
| N13 | - | - |
| N14 | - | - |
| N15 | - | - |
| N16 | - | - |
| N17 | - | - |
| N18 | - | - |
| N19 | - | - |
| N20 | - | - |
| N21 | - | + |
| N22 | - | - |
| N23 | - | - |
| N24 | - | - |
| N25 | - | - |
| N26 | - | - |
| N27 | - | - |
| N28 | - | - |
| N29 | - | - |
| N30 | - | - |
| N31 | + | - |
| N32 | - | - |
| N33 | - | - |
| N34 | - | - |
| N35 | - | - |
| N36 | - | - |
| N37 | + | - |
| N38 | - | - |
| N39 | - | - |
| N40 | - | - |
| N41 | - | - |
| N42 | - | - |
| N43 | - | - |
| N44 | - | - |
| N45 | - | - |
| N46 | + | + |
| N47 | - | - |
| N48 | - | - |
| N49 | - | - |
| N50 | - | - |
| N51 | - | - |
| N52 | - | - |
| N53 | - | - |
| N54 | - | - |
| N55 | - | - |
| N56 | - | - |
| N57 | - | - |
| N58 | - | - |
| N59 | - | - |
| N60 | - | - |
| N61 | - | - |
| N62 | - | - |
| N63 | + | - |
| N64 | - | - |
| N65 | - | - |
| N66 | - | + |
| N67 | - | - |
| N68 | - | - |
| N69 | - | + |
| N70 | - | + |
| N71 | - | - |
| N72 | - | - |
| N73 | - | - |
| N74 | - | - |
| N75 | - | - |
| N76 | - | - |
| N77 | - | - |
| N78 | - | - |
| N79 | - | - |
| N80 | - | - |
| N81 | - | - |
| N82 | - | + |
| N83 | - | - |
| N84 | - | - |
| N85 | - |  |
| N86 | - | + |
| N87 | - | - |
| N88 | - | - |
| N89 | - | - |
| N90 | - | - |
| N91 | - | - |
| N92 | - | - |
| N93 | - | + |
| N94 | - | - |
| N95 | - | - |
| N96 | - | - |
| N97 | - | - |
| N98 | - | - |
| N99 | + | - |
| N100 | - | - |
| N101 | - | - |
| N102 | - | - |
